# Digital fragment analysis of short tandem repeats by high‐throughput amplicon sequencing

**DOI:** 10.1002/ece3.2221

**Published:** 2016-06-08

**Authors:** Brian J. Darby, Shay F. Erickson, Samuel D. Hervey, Susan N. Ellis‐Felege

**Affiliations:** ^1^ Department of BiologyUniversity of North Dakota10 Cornell St. Stop 9019Grand ForksNorth Dakota58202

**Keywords:** Genotyping by sequencing, length homoplasy, marker, microsatellite, sequencing, short tandem repeat

## Abstract

High‐throughput sequencing has been proposed as a method to genotype microsatellites and overcome the four main technical drawbacks of capillary electrophoresis: amplification artifacts, imprecise sizing, length homoplasy, and limited multiplex capability. The objective of this project was to test a high‐throughput amplicon sequencing approach to fragment analysis of short tandem repeats and characterize its advantages and disadvantages against traditional capillary electrophoresis. We amplified and sequenced 12 muskrat microsatellite loci from 180 muskrat specimens and analyzed the sequencing data for precision of allele calling, propensity for amplification or sequencing artifacts, and for evidence of length homoplasy. Of the 294 total alleles, we detected by sequencing, only 164 alleles would have been detected by capillary electrophoresis as the remaining 130 alleles (44%) would have been hidden by length homoplasy. The ability to detect a greater number of unique alleles resulted in the ability to resolve greater population genetic structure. The primary advantages of fragment analysis by sequencing are the ability to precisely size fragments, resolve length homoplasy, multiplex many individuals and many loci into a single high‐throughput run, and compare data across projects and across laboratories (present and future) with minimal technical calibration. A significant disadvantage of fragment analysis by sequencing is that the method is only practical and cost‐effective when performed on batches of several hundred samples with multiple loci. Future work is needed to optimize throughput while minimizing costs and to update existing microsatellite allele calling and analysis programs to accommodate sequence‐aware microsatellite data.

## Introduction

Molecular ecology relies on the use of short tandem repeats (STR, or “microsatellites”) as neutral markers that can be useful in applications of forensic identification, genetic diversity, and population gene flow (Selkoe and Toonen 2006; Guichoux et al. 2011). Analysis of microsatellites traditionally involves PCR amplification of selected loci, using a primer containing a fluorescent dye, followed by capillary electrophoresis of PCR products along with a molecular “ladder” that serves to calibrate the length of products. Unfortunately, this method lends itself to numerous PCR amplification artifacts, imprecise sizing, nondetection of unique alleles due to length homoplasy, and limited ability to multiplex multiple loci per sample. High‐throughput sequencing has been proposed as a method to facilitate microsatellite analysis in both the discovery and genotyping phase (Guichoux et al. 2011). Initially, next‐generation sequencing was used to mine genomic shotgun or target capture sequences for the discovery of microsatellite loci (Malausa et al. 2011; Castoe et al. 2009; Abdelkrim et al. 2009). As sequencing reads grew in length, first with Ion Torrent semiconductor sequencer (Zhao et al. 2015; Zubakov et al. 2015; Fordyce et al. 2015), and now with MiSeq paired‐end sequencing by synthesis (Zeng et al. 2015), microsatellite amplicons can be sequenced through their tandem repeat region to the full length of the amplicon. The result of sequencing, after paired‐end merging and adapter trimming, is several million reads that are exactly the length of the microsatellite portion of the original PCR amplicon. This “digital” form of data is fundamentally different than the “analog” form of data collected by capillary electrophoresis, and it may prove to be a significant advance in the genotyping of microsatellite alleles (Børsting and Morling 2015). Several algorithms have been proposed to genotype microsatellites from sequencing data (Warshauer et al. 2013; Van Neste et al. 2014; Suez et al. 2016), and Suez et al. (2016) showed that sequencing data are quantitatively comparable to capillary electrophoresis data, but the question remains, “is it worth adopting the amplicon sequencing approach instead of capillary electrophoresis to genotype microsatellites?”

There are four main technical drawbacks of capillary electrophoresis that next‐generation sequencing may be able to solve: amplification artifacts, imprecise sizing, length homoplasy, and cost/multiplex capability. In our estimation, it would be advantageous to adopt amplicon sequencing to genotype microsatellites if this approach is found to have either a significant improvement in one of these drawbacks (artifacts, sizing, homoplasy, or cost), or moderate improvement in several drawbacks. Otherwise, it may instead be more beneficial for a laboratory to maintain the capillary electrophoresis approach to microsatellite genotyping. The objective of this project is to determine which, if any, of these drawbacks are improved by amplicon sequencing, and whether amplicon sequencing resulted in a moderate or significant improvement over capillary electrophoresis.

### Amplification artifacts

Polymerase chain reaction introduces numerous artifacts during amplification, such as slippage of the polymerase (which can alter the number of repeat units in a repetitive region) or incomplete extension during a cycle (which can cause chimeric amplicons formed by two heterologous templates). Additionally, *Taq* polymerase lacks 3′ to 5′ exonuclease proofreading activity and has a high error rate (which does not have a significant impact on analyzing microsatellites) and leaves 3′ dA overhangs on the ends of amplicons. Together, skipping of repeat units and incomplete extension of dA overhangs results in aberrant electrophoretic migration patterns like split or stutter peaks that can make it difficult to identify alleles correctly and consistently. PCR‐generated artifacts due to slippage will also be present in both capillary electrophoresis and sequencing data, but incomplete extension of dA overhangs are not a problem because they lie outside the range of what is sequenced. Even though the equivalent of split and stutter peaks are still present with sequencing, in theory it should be easier to discern the PCR artifacts because one would have the full sequence (and frequency) of all the reads and be able to reconstruct the history of artifact formation.

### Imprecise sizing

Slight variations in electrophoretic conditions, such as voltage, temperature, and polymer conditions, can alter the migration pattern and size estimates of the PCR fragments. Thus, identical fragments can appear to be different lengths when run on different machines or even different runs on the same machine. This introduces significant error rates within an experiment and limits the portability of data across different laboratories or projects. Perhaps the most significant advantage of fragment analysis by sequencing is that the sizing data used by the operator are digital, not analog. Digital sizing means that each nucleotide is sequenced, individually, and incrementally. Digital fragment analysis by sequencing removes the ambiguity that comes from trying to calibrate a PCR sample with a molecular ladder. Alleles are unambiguously called in whole number integer increments (e.g., 252 or 253 bp), whereas the analog capillary electrophoresis method often results in fractional lengths, such as “252.6 bp,” and the user may have to visually determine whether the true allele is 252 or 253 bp.

### Length homoplasy

Depending on the complexity of the microsatellite locus and its repeat structure, there may be nucleotide differences between alleles of the same length, called “length homoplasy,” that cannot be detected with capillary electrophoresis alone (Estoup et al. 2002). This limits the precision of analysis by reducing the true number of unique alleles called at each locus. A significant advantage of fragment analysis by sequencing is the ability to discern length homoplasy and resolve alleles of the same length but different repeat sequence. This is particularly helpful in loci with complicated structures and more than one adjacent repeat motif.

### Multiplex capability

Capillary electrophoresis has two main ways to multiplex a microsatellite assay and process multiple loci per sample: (1) label amplicons with contrasting fluorescent dyes, and (2) pool loci that are not expected to overlap in their lengths. For example, it would be possible to multiplex 12 different loci using four different dyes (FAM, VIC, NED, JOE) with three different length ranges, 100–200, 200–300, and 300–400. However, developing this level of multiplexing requires considerable testing and design effort and is only practical if one expects to process many samples for a long time. It is more common and more feasible to only multiplex four to six samples at a time. However, with fragment analysis by sequencing, there is no design limit to the number of loci that can be pooled and sequenced. The only design constraint is that the amplicons must be short enough so that the sequencing reads (currently at 300 bp in the case of MiSeq v3 chemistry) must at least extend past the repeat region so that the paired ends can be merged.

The objective of this project was to test a high‐throughput amplicon sequencing approach to fragment analysis of short tandem repeats and to characterize its advantages and disadvantages against traditional capillary electrophoresis. Most of the tests to date have been performed on a well‐tested human STR panel (Fordyce et al. 2015; Zeng et al. 2015). In molecular ecology and wildlife genetics, however, the more relevant challenge is to adapt microsatellite primer sets that have limited testing beyond their original application. To accomplish this, we redesigned existing primers that target 12 muskrat (*Ondatra zibethicus*) microsatellite loci to accommodate high‐throughput sequencing on the MiSeq Gene and Small Genome Sequencer. We amplified and sequenced all 12 loci for 180 muskrat specimens collected from North Dakota. We then analyzed the sequencing data for allele calling, propensity for amplification or sequencing artifacts, similarity to traditional capillary electrophoresis, evidence of length homoplasy, and finally a detailed cost analysis of the capillary electrophoresis versus the amplicon sequencing approach. Prior to this research, we perceived that digital fragment analysis by sequencing would be a valuable improvement over capillary electrophoresis if it improves on any of the current limitations (accuracy, precision, discrimination, throughput, and cost) without exacerbating other limitations or increasing the overall per‐sample cost.

## Methods

The primers of Laurence et al. (2009) were originally designed to accommodate multiplexing by capillary electrophoresis and therefore have a wide range of lengths, some exceeding 300 bp (the maximum length of a single MiSeq read). We used the cloned sequences they provided (Genbank accession numbers EU487259–EU487265 and EU999728–EU999733) to redesign primers that generate amplicons with a more uniform length distribution of around 150–275 bp and to include sequencing adapters on the 5′ end (Table S1). We also designed second‐round PCR primers to anneal to the 5′ end of the first‐round primers and include one of eight i5 indexes or one of 24 i7 indexes (Table S2) used to individually bar code each specimen.

**Figure 1:**
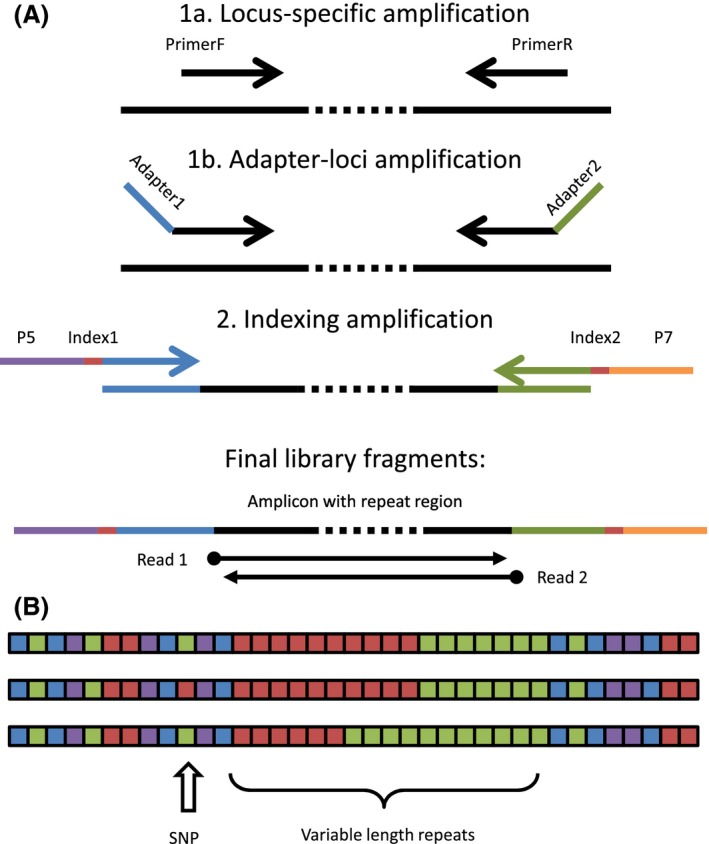
(A) Workflow of amplifying microsatellites for digital fragment analysis of short tandem repeats. Projects can begin with either locus‐specific amplification (1a, in multiplex, then 1b) or adapter‐loci amplification (1b, in singleplex). Loci are then pooled by sample and re‐amplified to integrate bar coding indexes (step 2) and sequenced with paired‐end reads sufficient to cross the repeat region (such as with Illumina MiSeq v3 chemistry). (B) Digital fragment analysis by sequencing can resolve two major types of sequence variation that lead to length homoplasy: single nucleotide polymorphisms (“SNP”), and variable length repeats of adjacent repeat motifs.

Muskrat specimens were collected from four main locations in eastern North Dakota (Tewaukon National Wildlife Refuge (NWR), Arrowwood NWR, Chase Lake NWR, and Devils Lake Basin, Fig. S1) as part of a larger muskrat ecology project in the state. Genomic DNA was extracted from 180 specimens (plus 12 nontissue blanks) by incubating 25 mg of liver tissue in genomic lysis buffer (1× DreamTaq PCR Buffer, 0.5% Tween, 0.5% Triton‐X, 100 ng/mL Protease *K*) at 60°C overnight followed by denaturing at 95°C for 15 min. Specimens were genotyped by amplicon sequencing using two rounds of PCR amplification (Fig. 1A). First‐round PCR was conducted in separate reactions for each locus (1× DreamTaq PCR buffer, 200 μm dNTPs, 0.2 μm each primer, 0.1 U DreamTaq polymerase, 2 μL DNA template) with an initial denaturation at 95°C (1 min) followed by six cycles of (30 sec at 95°C, 30 sec at 60°C decreasing by 1°C each cycle, 30 sec at 72°C), 24 cycles of (30 sec at 95°C, 30 sec at 60°C, 30 sec at 72°C), followed by a final extension at 72°C for 5 min. Ten microliter of each PCR product (across all loci from the same specimen) was pooled and cleaned using the Zymo PCR Cleanup kit, and 2 μL of the cleaned product was used as template for a second‐round PCR amplification using dual‐indexing primers (1× DreamTaq PCR buffer, 200 μm dNTPs, 0.1 μm each primer, 0.1 U DreamTaq polymerase, 2 μL DNA template) with an initial denaturation at 95°C (1 min) followed by eight cycles of 30 sec at (95°C, 30 sec at 55°C, 30 sec at 72°C), followed by a final extension at 72°C for 5 min. Five microliter from each specimen was pooled, cleaned using the Invitrogen Purelink PCR Cleanup kit, and submitted for sequencing on the MiSeq Gene & Small Genome Sequencer (MiSeq Reagent Kit v3, 2 × 300 bp reads).

**Figure 2:**
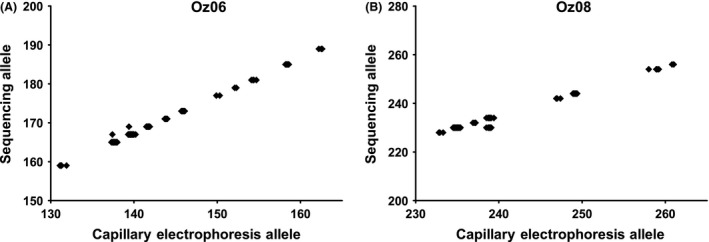
Comparison of microsatellite fragment analysis methods. Traditional capillary electrophoresis allele length (horizontal axis) is compared against amplicon sequencing allele length (vertical axis) for a subset of 90 specimens. (A) Microsatellite locus Oz06. (B) Microsatellite locus Oz08. In both loci, the sequencing primers (Table S1) were redesigned from the original primers intended for capillary electrophoresis (Laurence et al. 2009), so the sequencing alleles are 27 bases longer in Oz06 and 5 bases shorter in Oz08 than for capillary electrophoresis.

Demultiplexed paired‐end sequencing reads were merged into one read with Pear (Zhang et al. 2014), trimmed of adapter sequence with Cutadapt 1.8.3 (Martin 2011), and de‐replicated with USEARCH (Edgar 2010). A custom Python/Biopython script was used to sort reads by locus and count up the frequency of each unique read for each specimen. A text file with histograms (with sequence information) was created for each locus and used to visually genotype each specimen at each locus, according to traditional microsatellite allele‐calling principles as reviewed in Guichoux et al. (2011). These histograms are available as part of the supplementary data archive, and we estimate that it took fewer than 20 cumulative person‐hours to visually genotype all specimens for all 12 loci. Population structure was examined using program STRUCTURE v2.3.4 (Pritchard et al. 2000) by comparing two datasets: one with alleles representing length only (to mimic a dataset generated by traditional capillary electrophoresis), and the second with alleles informed with sequence data (which resolves length homoplasy). Both datasets were run with 50,000 burn‐in iterations followed by 100,000 measurement iterations for ten replicates each of *K* = 1–10 (assuming admixture model and correlated alleles). The results were evaluated with STRUCTURE HARVESTER (Earl and vonHoldt 2012) for optimal *K*‐value, and visualized with CLUMPAK (Kopelman et al. 2015). Deviations from Hardy–Weinberg Equilibrium were tested in GENEPOP (Raymond and Rousset 1995). Additionally, a subset of 90 specimens was genotyped at loci Oz06 and Oz08 by traditional capillary electrophoresis (Laurence et al. 2009). Finally, a cost analysis was performed to compare the estimated costs associated with traditional capillary electrophoresis to those of two alternative amplicon sequencing approaches: (1) a 2‐round PCR amplification (in which the first round begins with fusion primers amplified in singleplex for each locus, separately, as was performed in this study, beginning at step 1b. of Fig. 1A) or (2) a 3‐round PCR amplification (in which the first round begins with just the loci primers in multiplex, beginning at step 1a. of Fig. 1A). The cost estimates (Table S4) were based on typical reagent and consumables costs, plus in‐house sequencing and capillary electrophoresis costs (external service fees may be slightly higher).

## Results

### Sequencing output, coverage, and allele calling

Of 14.4 million quality‐filter passed reads, 10,789,866 reads were successfully trimmed and merged into one read and used for subsequent analysis. This resulted in a median of 55,767 reads per specimen and 3742 reads per specimen per locus. The largest ratio in read counts between the dominant allele to the subdominant allele was 16.0, with a median of 1.66 across all loci. All twelve nontissue blank samples were truly blank and had no apparent read signal. Twenty‐three specimens had missing data due to low coverage (fewer than 20 total reads) for just one or two loci (but kept in the dataset for analysis), while four specimens were removed from the dataset due to overall low coverage (fewer than 20 reads in the dominant allele across all loci). One locus (Oz30b) was removed from the analysis due to an indecipherable repeat pattern likely the result of chimeric PCR amplicons. This locus was commonly removed from analysis in two previous studies by the original developers (Laurence et al. 2011, 2013). In general, the length of alleles by amplicon sequencing was comparable to length of alleles as determined by capillary electrophoresis (Fig. 2). Other than in the cases of allelic dropout, the main practical difference between genotyping by amplicon sequencing and fragment analysis by capillary electrophoresis was that amplicon sequencing resulting in digital allele lengths (with single base‐pair resolution), whereas capillary electrophoresis resulted in analog allele lengths (of 0.1 base‐pair resolution).

**Figure 3:**
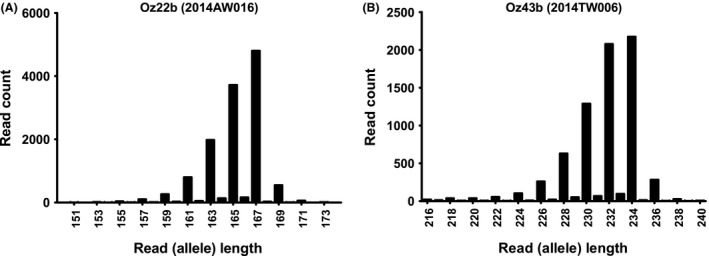
Frequency histograms of high‐throughput sequencing reads obtained for two loci (Oz22b and Oz43b) from two specimens (2014AW16 and 2014TW006, respectively). Both specimens would have been considered homozygous at these loci using fragment analysis by capillary electrophoresis, but fragment analysis by sequencing demonstrates heterozygous alleles due to length homoplasy (see Table 3).

A total of 294 different alleles were detected across 11 loci and 176 specimens using sequence data (Table 1, Table S3), and only 164 alleles would have been detected based on length only (e.g., by capillary electrophoresis), meaning about 44% of alleles would have been undetected using traditional methods. These cryptic alleles were detected as a result of two main types of variation: (1) variation in the length of different repeat motifs and (2) single nucleotide polymorphisms in the nonrepeating portion of the amplicon (Fig. 1B). For example, sequence data help to resolve length homoplasy in Oz22b (Fig. 3A). This locus has two adjacent repeat motifs (CT and CA), and fragment analysis by capillary electrophoresis would have suggested a homozygous 167/167 genotype for specimen AW016, but sequencing clearly demonstrated two different alleles of the same length but with contrasting repeat lengths (CT_16_/CA_14_ vs. CT_15_/CA_15_, Table 2). Thus, sequence data help to resolve length homoplasy as can be seen in Oz43b in specimen TW006 (Fig. 3B). This locus has a single nucleotide polymorphism at the fifth nucleotide after the primer, and sequencing clearly resolves this example of length homoplasy for what would have otherwise been considered homozygous (234/234, Table 2) or possibly heterozygous 232/234. Overall, Oz17b and Oz22b were the most allele‐rich loci, with seven alleles found in Oz22b of length 163, due to a combination of variations in both the length of individual repeat motifs and also polymorphisms outside of the repeat regions.

**Table 1:** Allelic richness (*A*), expected (*H*_*E*_), and observed heterozygosity (*H*_*O*_) for eleven muskrat microsatellite loci, comparing two datasets from the present study: length‐only (simulating the results of fragment analysis by capillary electrophoresis) and sequence‐aware (resulting from fragment analysis by sequencing). Asterisks indicate loci that deviated significantly from Hardy–Weinberg equilibrium

Locus	Length‐only data			Sequence‐aware data			Increase in alleles
	*A*	*H*_*E*_	*H*_*O*_	*A*	*H*_*E*_	*H*_*O*_	
Oz06b	14	0.789	0.807	19	0.880	0.898	+5 (1.4×)
Oz08b	9	0.629	0.602*	15	0.676	0.653	+6 (1.7×)
Oz16b	16	0.884	0.858	25	0.909	0.875	+9 (1.6×)
Oz17b	24	0.931	0.797*	69	0.957	0.826*	+45 (2.9×)
Oz22b	14	0.830	0.818	41	0.933	0.932	+27 (2.9×)
Oz27b	9	0.785	0.761	13	0.797	0.761	+4 (1.4×)
Oz32b	18	0.908	0.944*	25	0.912	0.944*	+7 (1.4×)
Oz34b	11	0.752	0.699	19	0.828	0.769*	+8 (1.7×)
Oz41b	18	0.876	0.845*	29	0.902	0.862*	+11 (1.6×)
Oz43b	19	0.904	0.879*	23	0.911	0.897*	+4 (1.2×)
Oz44b	12	0.698	0.737	16	0.738	0.777	+4 (1.3×)
Total:	164			294			+130 (1.8×)

**Table 2:** Fragment analysis by sequencing resolves length homoplasy due to sequence variations. Both parts of Table 2 (A and B) list the top ten most frequent reads for one locus of one specimen. (A) Specimen AW016 at locus Oz22b would have been considered homozygous 167/167 with capillary electrophoresis, but sequencing demonstrates that two different true alleles (167D and 167C) comprise the 167‐bp fragments due to a variable number of CT and CA repeats. (B) Specimen TW006 at locus Oz43b would have been considered homozygous 234/234 with capillary electrophoresis, but sequencing demonstrates that two different true alleles (234A and 234B) comprise the 234‐bp fragment due to a single nucleotide polymorphism (in bold font) outside of the repeat region

Rank	Length	Freq.	Sequence (part)	Interpretation
(A) Oz22b (specimen AW016), see corresponding Fig. 2A				
1	167	1477	… (CT)_16_(CA)_14_…	True allele 167D
2	167	1381	… (CT)_15_(CA)_15_…	True allele 167C
3	165	1154	… (CT)_15_(CA)_14_…	(−1, 0) stutter of 167D plus (0, −1) stutter of 167C
4	165	667	… (CT)_14_(CA)_15_…	Mostly (−1, 0) stutter of 167C
5	165	519	… (CT)_16_(CA)_13_…	Mostly (0, −2) stutter of 167D
6	163	505	… (CT)_14_(CA)_14_…	(−2, 0) stutter of 167D plus (−1, −1) stutter of 167C
7	163	409	… (CT)_15_(CA)_13_…	(−1, −1) stutter of 167D plus (0, −2) stutter of 167C
8	163	205	… (CT)_13_(CA)_15_…	Mostly (−2, 0) stutter of 167C
9	161	179	… (CT)_14_(CA)_13_…	Multiple stutters of both alleles
10	161	174	… (CT)_13_(CA)_14_…	Multiple stutters of both alleles
(B) Oz43b (specimen TW006), see corresponding Fig. 2B				
1	234	627	…GAGCACCTG**A**… (GT)_26_…	True allele 234A
2	232	590	…GAGCACCTG**A**… (GT)_25_…	(−1) stutter of 234A
3	234	522	…GAGCACCTG**C**… (GT)_26_…	True allele 234B
4	232	505	…GAGCACCTG**C**… (GT)_25_…	(−1) stutter of 234B
5	230	370	…GAGCACCTG**A**… (GT)_24_…	(−2) stutter of 234A
6	230	303	…GAGCACCTG**C**… (GT)_24_…	(−2) stutter of 234B
7	228	174	…GAGCACCTG**A**… (GT)_23_…	(−3) stutter of 234A
8	228	159	…GAGCACCTG**C**… (GT)_23_…	(−3) stutter of 234B
9	236	77	…GAGCACCTG**A**… (GT)_27_…	(+1) stutter of 234A
10	226	73	…GAGCACCTG**C**… (GT)_22_…	(−4) stutter of 234B

### Analysis of population structure and cost estimates

**Figure 4:**
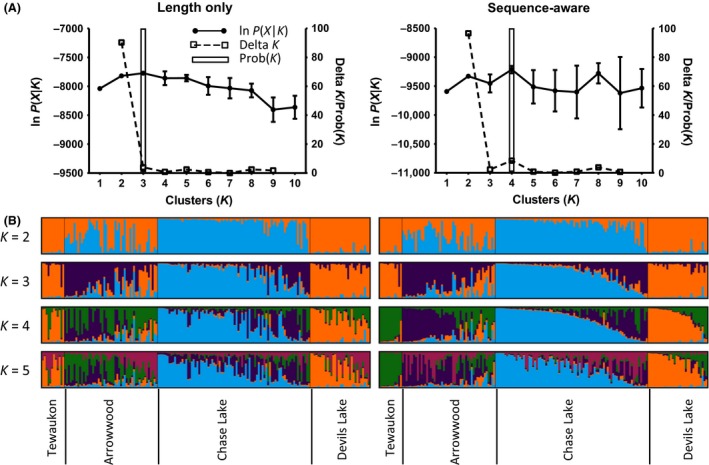
Cluster assignment of muskrat specimens from STRUCTURE analysis. (A) Plot of mean ln*P*(*X*|*K*), Delta *K*, and posterior probability *P*(*K*) against all ten test *K*‐values (*n* = 10 replicates per *K*‐value). (B) *Q*‐plots showing results from analysis of cluster number *K *=* *2 through 5. Data were analyzed twice: once using length‐only alleles, to simulate fragment analysis by capillary electrophoresis, and once using sequence‐aware allele to take advantage of fragment analysis by sequencing. Specimens are grouped by their original sampling location (Tewaukon NWR, Arrowwood NWR, Chase Lake NWR, and Devils Lake Basin). The different colors (blue, orange, purple, green, and pink) represent different clusters of genotypes. Each vertical line represents a different specimen, the color of which represents the likelihood of that specimen belong to the cluster. Specimens from the Tewaukon population were distinct from the Devils Lake population at *K *=* *4 clusters in the sequence‐aware dataset, but not in the length‐only dataset.

To determine whether the increased number of alleles would affect our ability to detect population genetic structure, we analyzed our data using both length‐only alleles (to mimic the data that would have been collected by capillary electrophoresis) and using sequencing‐aware alleles (the data that result from digital fragment analysis by sequencing and can resolve length homoplasy). All loci had greater expected and observed heterozygosity in the sequence‐aware dataset than with the length‐only dataset (Table 1). To evaluate the optimal number of clusters “*K*,” the “Delta *K*” method by Evanno et al. (2005) supported *K* = 2 for both populations (Fig. 4A). However, the posterior probability *P*(*K*) used by Pritchard et al. (2000) supports *K* = 3 populations for the length‐only dataset, but *K* = 4 populations for the sequence‐aware dataset (Fig. 4A). For *K* = 4, the sequence‐aware dataset discriminates the Tewaukon population from the Devils Lake population, while the length‐only dataset does not discriminate these two populations (Fig. 4B).

**Figure 5:**
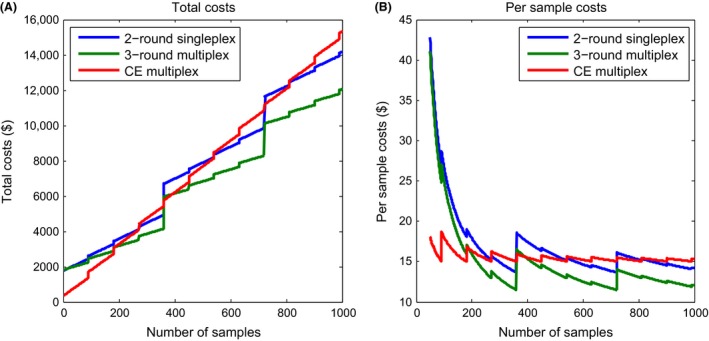
Cost analysis of microsatellite genotyping by traditional capillary electrophoresis versus two options of amplicon sequencing. All three strategies assume 12 microsatellite loci, and specific cost estimates for consumables and reagents are in Table S4. Traditional capillary electrophoresis (“CE multiplex”) is assumed to be performed in three batches per set of samples (4 loci per batch). Amplicon sequencing with two rounds of PCR (“2‐round Singlplex”) assumes the first round is performed in singleplex for each locus separately, beginning with step “1b” of Fig. 1A (as in this study). Amplicon sequencing with three rounds (“3‐round multiplex”) assumes that the first round is performed with all loci together in multiplex, beginning with step “1a” of Fig. 1A. Left: Total overall cost of entire project of up to 1000 samples, including sequencing (assuming in‐house costs). Right: Per‐sample costs of up to 1000 samples.

Research laboratories have different levels of access to equipment and discounts or bulk pricing on reagents and consumables, which makes it difficult to estimate per‐sample costs for either method. Nonetheless, we attempted a cost comparison between capillary electrophoresis and amplicon sequencing by making basic assumptions about the typical costs and workflow for three different genotyping approaches for 12 microsatellite loci: (1) capillary electrophoresis performed in three batches per set of samples (four loci per batch), (2) amplicon sequencing with two rounds of PCR (as in this study, where the first round is performed for each locus separately, beginning with step “1b” for Fig. 1A), and (3) amplicon sequencing with three rounds (where the first round is performed with all loci together in multiplex, beginning with step “1a” for Fig. 1A). For fewer than 192 samples (i.e., two 96‐well plates, including blanks), capillary electrophoresis generally is more cost‐effective than amplicon sequencing (Fig. 5). Greater than 192 samples, the lowest per‐sample cost depends on the approach and the degree to which the number of samples fills up a 96‐well plate and makes optimal use of a single sequencing run. However, the 3‐round PCR approach to amplicon sequencing consistently has lower per‐sample costs than the 2‐round approach. For 1000 samples, we predict that capillary electrophoresis will cost about $15 per sample, 2‐round singleplex amplicon sequencing will cost about $14 per sample, and 3‐round multiplex amplicon sequencing will cost about $12 per sample. For capillary electrophoresis, the highest costs are the enzymes, fluorescent dye‐labeled primers, and capillary electrophoresis runs. For amplicon sequencing, the highest costs are the enzymes, PCR cleanup kits, and the sequencing run. Thus, for fewer than 192 samples, capillary electrophoresis is probably more cost‐effective, especially if the dye‐labeled primers have already been purchased. For >192 samples, amplicon sequencing can result in a slightly lower cost per sample, depending on the research laboratory's access to equipment (in handling 96‐well plates), discounts in reagents and consumables, and whether or not fees are charged for sequencing.

## Discussion

The most significant finding of this work is that fragment analysis by sequencing not only detects a greater number of alleles than by capillary electrophoresis (Table 1, Fig. 3), but that this also appears to resolve a greater degree of population genetic structure (Fig. 4) and may reduce overall costs of projects with a large number of samples (Fig. 5). We would expect that the ability to detect a greater number of alleles will also improve many of the other applications of microsatellites, such as identifying subspecies delineations, detecting populations with low genetic diversity, and reconstructing pedigrees from wild populations. Even though PCR artifacts are still a problem in fragment analysis by sequencing (which show up as split or stutter peaks in capillary electrophoresis), with sequencing it is possible to interpret the nature of the PCR artifact based on the sequence of the amplicons (Table 2). Thus, the ability to resolve a greater number of alleles with increased precision is one of the most significant advantages of digital fragment analysis by sequencing rather than by capillary electrophoresis. High‐throughput sequencing is now also being used to genotyping single nucleotide polymorphisms (Davey and Blaxter 2010), in which sequencing reads cover a focused subset of the genome (and a portion of these reads contain informative SNPs). However, sequencing libraries for this method are prepared by first digesting template DNA with a restriction enzyme, then by amplifying adapter‐ligated fragments. We believe that there will be a continued need for genotyping microsatellites in at least two key circumstances: (1) to maintain consistency with previously collected data and (2) when the template DNA is of low quality or low quantity and demands that the first molecular step be PCR amplification rather than restriction digestions, such as with ancient or degraded DNA, or DNA from challenging samples such as bone, hairs, or collected on FTA cards.

However, the increased number of alleles also raises the complication of allele nomenclature, reporting, and even analysis in some currently used software programs. Gelardi et al. (2014) proposed a change in STR allele nomenclature to explicitly include both the repeat sequence and the repeat number. We agree that this could be useful for well‐tested loci with simple repeat motifs. However, for many species of ecological interest, the microsatellite loci will have minimal testing and often complex repeat motifs, such as the muskrat microsatellites used in this study. For these circumstances, allele names would need to reflect both variations in the length of individual repeat motifs and also polymorphisms outside of the repeat regions, and that could result in an unwieldy nomenclature. To ensure forwards and backwards compatibility, and until a consensus nomenclature is established for sequence‐aware microsatellite loci, we recommend that all studies publish (1) the entire sequence of all alleles detected and (2) a unique allele name that reflects both the length of the allele and an identifier that is unique at least within the study (see Table S3).

One convenience of fragment analysis by sequencing is that it is very compatible with existing microsatellite loci workflows with only a few additional oligonucleotides to purchase and, in most cases, few additional primers to develop. The primary criterion for fragment analysis by sequencing is that the sequencing read must fully cover the entire length of all alleles. In the case of paired‐end sequencing such as Illumina MiSeq v3 chemistry (with 2 × 300 bp reads) this means that the paired reads must overlap unambiguously with each other *outside of* the internal repeat region (i.e., reads that end *inside of* a repeat region cannot be merged accurately). This works best if all alleles are expected to be <300 bp long (locus‐specific primers included). Most microsatellite loci already fit this criteria or can be redesigned using sequence data surrounding the repeat region. Thus, it is important for forward compatibility that newly developed microsatellite primers should also report the full sequence of nucleotides surrounding the tandem repeat region so that primers can be modified to suit various needs. Sequencing technology with longer read lengths would be a benefit here, but it is also possible that some microsatellites with long repeat regions may not be compatible with this approach. It will also be necessary to modify the existing allele‐calling algorithms to be able to combine sequence data with read frequency to discriminate PCR artifacts from true alleles, as well as statistics programs that detect allelic dropout. Many of these programs make an explicit assumption that alleles are separated by the length of their longest repeat unit (e.g., Suez et al. 2016), but this is not necessarily the case (e.g., see alleles 234A and 234B for locus Oz43b, Table 2B). At the moment there is no sequence‐aware allele‐calling program that we are aware of, so visual allele calling is a current limitation of this approach. However, we anticipate that automated, sequence‐aware allele‐calling programs will become available as this method becomes more common. Furthermore, there is a need for both theoretical analysis and software development to accommodate sequence‐aware alleles, which may pose a difficulty in the context of the traditional stepwise mutation model used to analyze microsatellites in many programs. However, the computational challenge of genotyping microsatellites from chromatogram peaks, and the software algorithms to analyze population genetic data, were quickly solved by the scientific community when microsatellites where performed by capillary electrophoresis. We expect that they will be solved just as quickly for microsatellites that are genotyped by sequencing.

Additional work is needed to determine the optimal workflow to increase throughput and decrease costs while maintaining adequate coverage and accuracy. Although this method has the potential for moderate cost savings, these savings depend on the organization of the project. For example, our study involved two rounds of PCR: the first with fusion primers containing both the locus‐specific annealing site and the sequencing adapter sites (step 1b of Fig. 1A), and the second round with adapter primers that have the sequencing adapter, bar coding indexes, and flow cell adapter sequences (step 2 of Fig. 1A). The first‐round PCR was performed in singleplex reactions because we found that multiplex reactions with lengthy (50+ bp) primers were problematic and produced length dimers and hairpin artifacts regardless of reaction conditions. An alternative, multiplexed, approach may be to use three rounds of PCR where the first round is multiplexed amplification using just loci‐specific primers (step 1a of Fig. 1A), then re‐amplify with pooled multiplex adapter primers (step 1b of Fig. 1A). A challenge of multiplexed amplification is the formation of chimeric reads consisting of multiple loci in one amplicon. These artifacts would be detectable by analyzing the sequences, but they would also lower the overall effective per run read output and may increase the number of failed genotypes or the number of specimens that need to be rerun due to low coverage. There is always a trade‐off between multiplexing and coverage. Multiplexing many samples and loci together lowers the overall per locus cost, but also results in lower coverage per locus, and an increased likelihood that a sample or locus does not get genotyped. Our current application required the use of the MiSeq sequencing platform in order to accommodate alleles longer than 250 bp. However, for short alleles that would be covered by paired‐end 2 × 150 bp reads, it may be possible to use a platform with much higher read output (such as the HiSeq platform) and obtain billions of reads instead of millions. In this case, one could multiplex many more samples or loci together in the same run and still achieve adequate coverage of each locus. A significant limitation of this method, though, is that repeating failed reactions require an additional whole sequencing run (or pooling with an existing batch), whereas for capillary electrophoresis it is fairly easy to rerun failed reactions. Researchers that genotype microsatellites by sequencing will need to build in room in their sequencing batches for reruns of failed samples. It is also important to note that samples from one species can be sequenced alongside samples from another species, which might make it easier to complete projects by coordinating sequencing runs with other projects or researchers.

In conclusion, we found that digital fragment analysis of short tandem repeats by high‐throughput sequencing was more accurate, precise, and cost‐effective than conventional capillary gel electrophoresis. We were able to (1) discern cryptic alleles that would have been hidden by length homoplasy, (2) have better internal consistency on sizing, and (3) process more samples at a lower cost using next‐generation sequencing instead of capillary electrophoresis. The primary advantages of fragment analysis by sequencing are the ability to size fragments precisely, resolve length homoplasy, multiplex many individuals and many loci into a single high‐throughput run, and compare data across projects and across laboratories (present and future) with minimal technical calibration (Table 3). A significant disadvantage of fragment analysis by sequencing is that the method is only practical and cost‐effective when performed on batches of several hundred samples with multiple loci or in collaboration with other species or projects. Thus, we recommend that researchers (or users of microsatellite loci) consider genotyping by amplicon sequencing especially if they have several hundred samples or complex loci with multiple repeating motifs. Projects with fewer than a couple hundred samples, or simple loci with a single repeat motif, may not benefit as much from genotyping by amplicon sequencing.

**Table 3:** Current status of fragment analysis by capillary electrophoresis and by sequencing

Feature	Capillary electrophoresis	Sequencing	Improvement
Split/stutter peaks	Present, ambiguous	Present, interpretable	No change
dA overhang	Present, minor problem	Absent, no problem	Moderate
Sizing	Imprecise, analog	Precise, digital	Moderate
Length homoplasy	Not discernible	Discernible	Significant
Multiplexing	Limited by length overlap and available dyes	Limited by desired coverage and bar coding capacity	Moderate
Interlaboratory portability	Poor	Excellent	Significant
Throughput	Low, Medium	Medium, High	Moderate
Cost^a^	$15	$12–14	Moderate
Allele nomenclature	Simple, based on length	Not simple, not standardized	Work needed
Allele‐calling software	Many	Few^b^	Work needed

a Per‐sample costs, depending on multiplexing design and costs of consumables and sequencing services.

b But see Warshauer et al. (2013) and Van Neste et al. (2014) and Suez et al. 2016.

## Data Accessibility

The following data and archives are available from DRYAD [DRYAD entry doi:10.5061/dryad.68mc8

1. Original sequencing files (FASTQ format, with a list of primers and metadata to map samples to specimens).
2. Histogram files used to manually genotype each specimen (one text file per locus).
3. Final genotypes (text file with metadata mapping allele to sequence).

## Conflict of Interest

None declared.

## Supporting information


**Figure S1**. Map of major physiographic regions of North Dakota with the four primary field sites from which muskrat specimens were obtained (one PNG image).

**Table S1**. First Round Oligos used for each of 12 loci (one tab‐delimited text file).

**Table S2**. Second Round Oligos used to index 16 × 12 = 192 samples (one tab‐delimited text file).

**Table S3**. Unique Alleles identified through sequencing (one tab‐delimited text file).

**Table S4**. Assumptions of per‐item cost and usage in the cost comparison analysis (one Excel file).
