# Supplementary figures and images for: Digital fragment analysis of short tandem repeats by high‐throughput amplicon sequencing
Source: Ecol Evol. 2016 Jun 8;6(13):4502–12. doi: 10.1002/ece3.2221 (PMC4930997; doi:10.1002/ece3.2221)

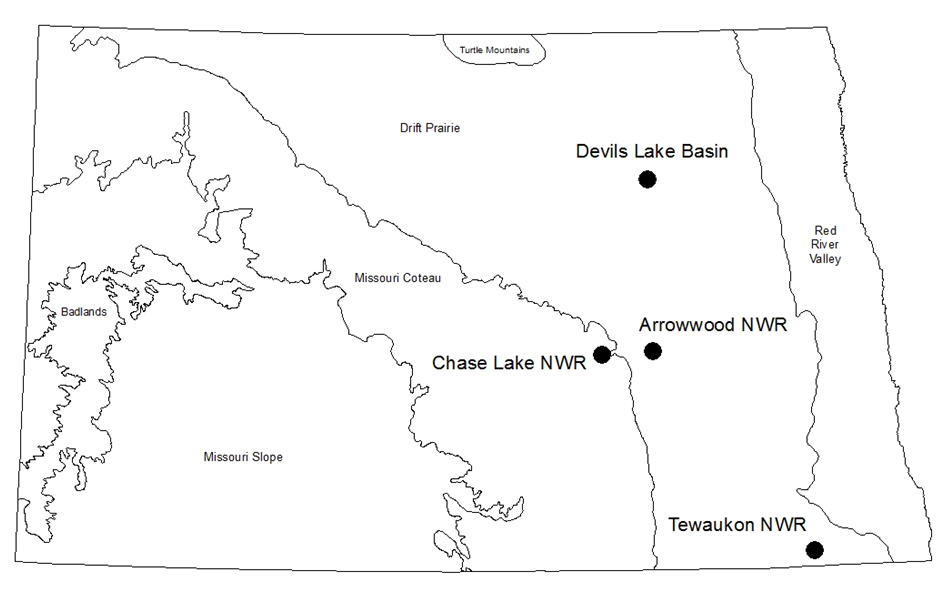

Supplement: Supplementary file 1 — Figure S1. Map of major physiographic regions of North Dakota with the four primary field sites from which muskrat specimens were obtained (one PNG image). [file ECE3-6-4502-s001.png]
