# Supplementary material for: Digital fragment analysis of short tandem repeats by high‐throughput amplicon sequencing
Source: Ecol Evol. 2016 Jun 8;6(13):4502–12. doi: 10.1002/ece3.2221 (PMC4930997; doi:10.1002/ece3.2221)
Supplement: Supplementary file 2 — Table S1. First Round Oligos used for each of 12 loci (one tab‐delimited text file). [file ECE3-6-4502-s002.docx]

|  | Table S1. Oligos used in first-round amplification |  |  |
| --- | --- | --- | --- |
|  |  |  |  |
| ID: | Sequence (5' to 3'): | Locus: | Side: |
| Oz06bF | TCG TCG GCA GCG TCA GAT GTG TAT AAG AGA CAG GGA CGG TCA CTC AGG AGA AA | Oz06b | Forward (i5) |
| Oz06bR | GTC TCG TGG GCT CGG AGA TGT GTA TAA GAG ACA GTT GTA AGA AGC CTG CTG ACA | Oz06b | Reverse (i7) |
| Oz08bF | TCG TCG GCA GCG TCA GAT GTG TAT AAG AGA CAG TAT GGG ACT GAC GGC TAA GG | Oz08b | Forward (i5) |
| Oz08bR | GTC TCG TGG GCT CGG AGA TGT GTA TAA GAG ACA GGG ACT CTG CCT TGA TGA CCT | Oz08b | Reverse (i7) |
| Oz16bF | TCG TCG GCA GCG TCA GAT GTG TAT AAG AGA CAG CTG TTC CAC ACA CAT GCA CA | Oz16b | Forward (i5) |
| Oz16bR | GTC TCG TGG GCT CGG AGA TGT GTA TAA GAG ACA GTT GCT CTT CCT GGT GTC TGG | Oz16b | Reverse (i7) |
| Oz17bF | TCG TCG GCA GCG TCA GAT GTG TAT AAG AGA CAG AGT CTG GAA TCT TTG CAA GGC | Oz17b | Forward (i5) |
| Oz17bR | GTC TCG TGG GCT CGG AGA TGT GTA TAA GAG ACA GTG CCA TGT TTT CTG GTT GGG | Oz17b | Reverse (i7) |
| Oz22bF | TCG TCG GCA GCG TCA GAT GTG TAT AAG AGA CAG CAT CCT GCG TCC TGT CTT TG | Oz22b | Forward (i5) |
| Oz22bR | GTC TCG TGG GCT CGG AGA TGT GTA TAA GAG ACA GGT GGT AAA AGT TGC TGG CCA | Oz22b | Reverse (i7) |
| Oz27bF | TCG TCG GCA GCG TCA GAT GTG TAT AAG AGA CAG TGG CTG AAA TGA AAC TGG CT | Oz27b | Forward (i5) |
| Oz27bR | GTC TCG TGG GCT CGG AGA TGT GTA TAA GAG ACA GTC TGA ACT GGT GTG GGA TTG T | Oz27b | Reverse (i7) |
| Oz30bF | TCG TCG GCA GCG TCA GAT GTG TAT AAG AGA CAG CGC CTT ATC TTT GCT TCG GT | Oz30b | Forward (i5) |
| Oz30bR | GTC TCG TGG GCT CGG AGA TGT GTA TAA GAG ACA GTT TTC GTG GCT GAA TAC CCA | Oz30b | Reverse (i7) |
| Oz32bF | TCG TCG GCA GCG TCA GAT GTG TAT AAG AGA CAG TGA GTG CTA CCA TAT GTT CCT CA | Oz32b | Forward (i5) |
| Oz32bR | GTC TCG TGG GCT CGG AGA TGT GTA TAA GAG ACA GTG CAA TTC TGT GGC TAG GAA C | Oz32b | Reverse (i7) |
| Oz34bF | TCG TCG GCA GCG TCA GAT GTG TAT AAG AGA CAG CAA CAT TGT GTA CTG AGC AAC T | Oz34b | Forward (i5) |
| Oz34bR | GTC TCG TGG GCT CGG AGA TGT GTA TAA GAG ACA GGA TTC TCT CTC TTT CAC TCA T | Oz34b | Reverse (i7) |
| Oz41bF | TCG TCG GCA GCG TCA GAT GTG TAT AAG AGA CAG TGA CAT TGA CCC AGG GAA GT | Oz41b | Forward (i5) |
| Oz41bR | GTC TCG TGG GCT CGG AGA TGT GTA TAA GAG ACA GTG ACG GTG CAG ATT TGG TTC | Oz41b | Reverse (i7) |
| Oz43bF | TCG TCG GCA GCG TCA GAT GTG TAT AAG AGA CAG TGA ACA GAA GGG AGT GAG CA | Oz43b | Forward (i5) |
| Oz43bR | GTC TCG TGG GCT CGG AGA TGT GTA TAA GAG ACA GCC CCT CAA TCA CAC AAG CAC | Oz43b | Reverse (i7) |
| Oz44bF | TCG TCG GCA GCG TCA GAT GTG TAT AAG AGA CAG CCA GAG AGG TTA CCG AAA TGA | Oz44b | Forward (i5) |
| Oz44bR | GTC TCG TGG GCT CGG AGA TGT GTA TAA GAG ACA GCC ACA CGC AGG AAA CAC ATT | Oz44b | Reverse (i7) |
|  |  |  |  |
|  |  |  |  |
