# Supplementary material for: Digital fragment analysis of short tandem repeats by high‐throughput amplicon sequencing
Source: Ecol Evol. 2016 Jun 8;6(13):4502–12. doi: 10.1002/ece3.2221 (PMC4930997; doi:10.1002/ece3.2221)
Supplement: Supplementary file 3 — Table S2. Second Round Oligos used to index 16 × 12 = 192 samples (one tab‐delimited text file). [file ECE3-6-4502-s003.docx]

|  | Table S2. Oligos used in Second-round PCR |  |  |
| --- | --- | --- | --- |
|  |  |  |  |
| ID: | Sequence (5' to 3'): | Index to use for data entry sheet | Side: |
| S502 | AAT GAT ACG GCG ACC ACC GAG ATC TAC ACC TCT CTA TTC GTC GGC AGC GTC | CTCTCTAT | Forward (i5) |
| S503 | AAT GAT ACG GCG ACC ACC GAG ATC TAC ACT ATC CTC TTC GTC GGC AGC GTC | TATCCTCT | Forward (i5) |
| S505 | AAT GAT ACG GCG ACC ACC GAG ATC TAC ACG TAA GGA GTC GTC GGC AGC GTC | GTAAGGAG | Forward (i5) |
| S506 | AAT GAT ACG GCG ACC ACC GAG ATC TAC ACA CTG CAT ATC GTC GGC AGC GTC | ACTGCATA | Forward (i5) |
| S507 | AAT GAT ACG GCG ACC ACC GAG ATC TAC ACA AGG AGT ATC GTC GGC AGC GTC | AAGGAGTA | Forward (i5) |
| S508 | AAT GAT ACG GCG ACC ACC GAG ATC TAC ACC TAA GCC TTC GTC GGC AGC GTC | CTAAGCCT | Forward (i5) |
| S510 | AAT GAT ACG GCG ACC ACC GAG ATC TAC ACC GTC TAA TTC GTC GGC AGC GTC | CGTCTAAT | Forward (i5) |
| S511 | AAT GAT ACG GCG ACC ACC GAG ATC TAC ACT CTC TCC GTC GTC GGC AGC GTC | TCTCTCCG | Forward (i5) |
| S513 | AAT GAT ACG GCG ACC ACC GAG ATC TAC ACT CGA CTA GTC GTC GGC AGC GTC | TCGACTAG | Forward (i5) |
| S515 | AAT GAT ACG GCG ACC ACC GAG ATC TAC ACT TCT AGC TTC GTC GGC AGC GTC | TTCTAGCT | Forward (i5) |
| S516 | AAT GAT ACG GCG ACC ACC GAG ATC TAC ACC CTA GAG TTC GTC GGC AGC GTC | CCTAGAGT | Forward (i5) |
| S517 | AAT GAT ACG GCG ACC ACC GAG ATC TAC ACG CGT AAG ATC GTC GGC AGC GTC | GCGTAAGA | Forward (i5) |
| S518 | AAT GAT ACG GCG ACC ACC GAG ATC TAC ACC TAT TAA GTC GTC GGC AGC GTC | CTATTAAG | Forward (i5) |
| S520 | AAT GAT ACG GCG ACC ACC GAG ATC TAC ACA AGG CTA TTC GTC GGC AGC GTC | AAGGCTAT | Forward (i5) |
| S521 | AAT GAT ACG GCG ACC ACC GAG ATC TAC ACG AGC CTT ATC GTC GGC AGC GTC | GAGCCTTA | Forward (i5) |
| S522 | AAT GAT ACG GCG ACC ACC GAG ATC TAC ACT TAT GCG ATC GTC GGC AGC GTC | TTATGCGA | Forward (i5) |
| N701 | CAA GCA GAA GAC GGC ATA CGA GAT TCG CCT TAG TCT CGT GGG CTC GG | TAAGGCGA | Reverse (i7) |
| N702 | CAA GCA GAA GAC GGC ATA CGA GAT CTA GTA CGG TCT CGT GGG CTC GG | CGTACTAG | Reverse (i7) |
| N703 | CAA GCA GAA GAC GGC ATA CGA GAT TTC TGC CTG TCT CGT GGG CTC GG | AGGCAGAA | Reverse (i7) |
| N704 | CAA GCA GAA GAC GGC ATA CGA GAT GCT CAG GAG TCT CGT GGG CTC GG | TCCTGAGC | Reverse (i7) |
| N705 | CAA GCA GAA GAC GGC ATA CGA GAT AGG AGT CCG TCT CGT GGG CTC GG | GGACTCCT | Reverse (i7) |
| N706 | CAA GCA GAA GAC GGC ATA CGA GAT CAT GCC TAG TCT CGT GGG CTC GG | TAGGCATG | Reverse (i7) |
| N707 | CAA GCA GAA GAC GGC ATA CGA GAT GTA GAG AGG TCT CGT GGG CTC GG | CTCTCTAC | Reverse (i7) |
| N710 | CAA GCA GAA GAC GGC ATA CGA GAT CAG CCT CGG TCT CGT GGG CTC GG | CGAGGCTG | Reverse (i7) |
| N711 | CAA GCA GAA GAC GGC ATA CGA GAT TGC CTC TTG TCT CGT GGG CTC GG | AAGAGGCA | Reverse (i7) |
| N712 | CAA GCA GAA GAC GGC ATA CGA GAT TCC TCT ACG TCT CGT GGG CTC GG | GTAGAGGA | Reverse (i7) |
| N714 | CAA GCA GAA GAC GGC ATA CGA GAT TCA TGA GCG TCT CGT GGG CTC GG | GCTCATGA | Reverse (i7) |
| N715 | CAA GCA GAA GAC GGC ATA CGA GAT CCT GAG ATG TCT CGT GGG CTC GG | ATCTCAGG | Reverse (i7) |
|  |  |  |  |
|  |  |  |  |
